# Prevalence and mortality of ceftazidime/avibactam-resistant KPC-producing *Klebsiella pneumoniae* bloodstream infections (2018–2022)

**DOI:** 10.1007/s10096-023-04712-8

**Published:** 2023-11-21

**Authors:** Matteo Boattini, Gabriele Bianco, Paulo Bastos, Sara Comini, Silvia Corcione, André Almeida, Cristina Costa, Francesco Giuseppe De Rosa, Rossana Cavallo

**Affiliations:** 1 Microbiology and Virology Unit, University Hospital Città Della Salute E Della Scienza Di Torino, Corso Bramante 88/90, 10126 Turin, Italy; 2 Department of Public Health and Paediatrics, University of Torino, Turin, Italy; 3 Lisbon Academic Medical Centre, Lisbon, Portugal; 4 CEDOC, Chronic Diseases Research Centre, NOVA Medical School, Lisbon, Portugal; 5 Department of Medical Sciences, Infectious Diseases, University of Turin, 10126 Turin, Italy; 6 Department of Internal Medicine 4, Hospital de Santa Marta, Central Lisbon Hospital Centre, Lisbon, Portugal; 7 NOVA Medical School, Universidade Nova de Lisboa, Campo Dos Mártires da Pátria 130, 1169-056 Lisbon, Portugal; 8 Unit of Infectious Diseases, Cardinal Massaia, 14100 Asti, Italy

**Keywords:** KPC, Bloodstream infection, Ceftazidime/avibactam resistance, *Klebsiella pneumoniae*, Sepsis, Mortality

## Abstract

**Introduction:**

Ceftazidime/avibactam-resistance in *Klebsiella pneumoniae* carbapenemase-producing *Klebsiella pneumoniae* (KPC-Kp) is a topic of great interest for epidemiological, diagnostic, and therapeutical reasons. However, data on its prevalence and burden on mortality in patients with bloodstream infection (BSI) are lacking. This study was aimed at identifying risk factors for mortality in patients suffering from ceftazidime/avibactam-resistant KPC-Kp BSI.

**Methods:**

An observational retrospective study (January 2018–December 2022) was conducted at a tertiary hospital including all consecutive hospitalized adult patients with a ceftazidime/avibactam-resistant KPC-Kp BSI. Data on baseline clinical features, management, and admission outcomes were analyzed.

**Results:**

Over the study period, among all the KPC-Kp BSI events recorded, 38 (10.5%) were caused by ceftazidime/avibactam-resistant KPC-Kp strains, 37 events being finally included. The ceftazidime/avibactam-resistant KPC-Kp strains revealed susceptibility restoration to at least one carbapenem in more than 60% of cases. In-hospital and 30-day all-cause mortality rates were 22% and 16.2%, respectively. Non-survivors suffered from more baseline comorbidities and experienced a more severe ceftazidime/avibactam-resistant KPC-Kp BSI presentation (i.e., both the Pitt Bacteremia and INCREMENT-CPE scores were significantly higher). Presenting with a higher Charlson Comorbidity Index, chronic kidney disease—KDIGO stage 3A or worse—having recently gone through renal replacement therapy, having suffered from an acute kidney injury following the ceftazidime/avibactam-resistant KPC-Kp BSI, and being admitted for cardiac surgery were the strongest predictors of mortality.

**Conclusion:**

Ceftazidime/avibactam resistance in KPC-Kp BSI easily emerged in our highly KPC-Kp endemic area with remarkable mortality rates. Our findings might provide physicians possibly actionable information when managing patients with a ceftazidime/avibactam-resistant KPC-Kp BSI.

**Supplementary Information:**

The online version contains supplementary material available at 10.1007/s10096-023-04712-8.

## Introduction

*Klebsiella pneumoniae* carbapenemase-producing *Klebsiella pneumoniae* (KPC-Kp) emerged globally as one of the most clinically relevant pathogen in view of its wide dissemination in healthcare facilities, the limited number of effective treatment options, and the resulting high mortality rates of the associated infections [1]. The alert issued in Italy based on data from 2014–2017 highlighted the endemic burden of KPC-Kp often characterized by a relevant number of bloodstream infections (BSIs) and endangering patient safety [2]. In 2018, the novel β-lactam/β-lactamase inhibitor combination ceftazidime/avibactam was introduced into clinical practice having shown excellent clinical activity against KPC-Kp [3–9]. More recently, it has been reported as the only independent survival predictor among KPC-Kp BSI patients [6, 9]. However, 5 years following the onset of clinical use, in vivo selection events of ceftazidime/avibactam-resistant strains [10, 11] and nosocomial outbreaks of ceftazidime/avibactam-resistant KPC-Kp have been reported [12–14]. Acquired resistance to ceftazidime/avibactam seems to be mainly due to amino acid substitutions in β-lactamases, alterations of ompK35/36 porins, and/or overexpression of efflux pumps. At the present time, from an epidemiological point of view, the most common resistance mechanism is the expression of KPC variants consisting of single amino acid substitutions between the positions 164–179 in the Ω loop region (most notably the Asp179Tyr—D179Y substitution). These variants are characterized by the loss of carbapenemase activity and the restoration of susceptibility to carbapenems, together with a concomitant diminished binding to avibactam. Other KPC variants encode mutations outside the Ω loop region, not necessarily having established a relationship with a previous ceftazidime/avibactam treatment [15–19]. Notably, while molecular testing is capable of detecting all KPC mutants, phenotypic and immunochromatographic methods revealed significant issues regarding the detection of KPC variants with diminished carbapenemase activity [19–22]. Moreover, strains harboring specific KPC mutants have been shown to have co-resistance towards both ceftazidime/avibactam and the recently approved cefiderocol [23, 24], meropenem-vaborbactam, and imipenem-relebactam [25]. Altogether, the spread of the KPC enzyme bearing high evolutionary potential, the limitations of diagnostic tests resulting in missed infection control practices, and the acquisition of resistance to newly introduced drugs might be paving the way to widespread waves of antimicrobial resistance. Ceftazidime/avibactam-resistant KPC-Kp strains are a topic of great interest and have been well described from an evolutionary point of view [26, 27], but data on their prevalence in KPC-Kp BSI and burden on mortality are lacking. We have sought to retrospectively pinpoint clinical features of relevance for mortality in patients with ceftazidime/avibactam-resistant KPC-Kp BSI in a high KPC-Kp endemicity setting over a 5-year period starting from the introduction of ceftazidime/avibactam into clinical practice. Accordingly, the present study was aimed at identifying risk factors for in-hospital and 30-day all-cause mortality to provide microbiologists and physicians with prognostic and possibly actionable information when managing patients with a ceftazidime/avibactam-resistant KPC-Kp BSI.

## Material and methods

### Study design

This observational retrospective study was conducted from January 1 2018 to December 31 2022 in Azienda Ospedaliero-Universitaria “Città della Salute e della Scienza di Torino” (Turin, Italy), one of the largest health center in Europe with a total bed capacity of 1900. All consecutive adult patients hospitalized due to ceftazidime/avibactam-resistant KPC-Kp BSI were included. Patient electronic medical records were used for collecting the following data: patient baseline clinical characteristics including Charlson Comorbidity Index, reason for admission, source of the ceftazidime/avibactam-resistant KPC-Kp BSI, ceftazidime/avibactam-resistant KPC-Kp BSI severity including Pitt Bacteremia score and INCREMENT-CPE score, ceftazidime/avibactam-resistant KPC-Kp antimicrobial susceptibility testing results, ceftazidime/avibactam-resistant KPC-Kp BSI target antibiotic therapy, and admission outcomes/complications.

### Definitions

Blood culture (BC) positivity for a ceftazidime/avibactam-resistant KPC-Kp strain and concomitant systemic inflammatory response syndrome signs were the criteria used to define a ceftazidime/avibactam-resistant KPC-Kp BSI episode. The ceftazidime/avibactam-resistant KPC-Kp BSI onset was defined as the date of the index positive BC was performed. The National Healthcare Safety Network criteria were used to define the source of the ceftazidime/avibactam-resistant KPC-Kp BSI, primary ceftazidime/avibactam-resistant KPC-Kp BSI being defined as not secondary to infection at another body site [28]. Active antibiotic therapy was defined when started within 48 h of the ceftazidime/avibactam-resistant KPC-Kp BSI onset and when the ceftazidime/avibactam-resistant KPC-Kp strain was susceptible in vitro to at least one prescribed drug. Antibiotic combination therapy included at least one other antimicrobial administered for ≥ 48 h. Prolonged infusion of antibiotic was defined as an antibiotic dose administered over a period of at least 2 h. Antibiotic dose adjusted for impaired renal function was defined as EUCAST dose [29] reduction of renally cleared antibiotics in patients with impaired renal function. Superimposed and/or following the ceftazidime/avibactam-resistant KPC-Kp BSI treatment candidemia was defined as a BSI event documented by BC positivity for a *Candida* spp. strain during or following the ceftazidime/avibactam-resistant KPC-Kp BSI treatment. KPC-Kp infection relapse was defined as the onset of a second microbiologically documented KPC-Kp infection in a patient whose original ceftazidime/avibactam-resistant KPC-Kp BSI had been clinically cured with resolution of symptoms/signs and negative BCs. Ceftazidime/avibactam exposure was defined as documented treatment with ceftazidime/avibactam for more than 72 h in 90 days preceding the ceftazidime/avibactam-resistant KPC-Kp BSI.

### Microbiological diagnostics

The BACT/ALERT Virtuo (bioMérieux, Marcy l’Ètoile, France) and BACT/ALERT FA and FN Plus BCs bottles (bioMérieux, Marcy l’Ètoile, France) were the BCs detection system and bottles used during this study, respectively. Positive BCs were subjected to Gram staining and subculture on appropriate solid medium. MALDI-TOF analysis (Bruker DALTONIK GmbH, Bremen, Germany) and Xpert Carba-R on GeneXpert platform (Cepheid, Sunnyvale, CA) were used for species identification and detection of KPC production in *K*. *pneumoniae* isolates, respectively. Susceptibility to major antimicrobials was performed using a commercial microdilution system (Panel NMDR, MicroScan WalkAway 96 Plus, Beckman Coulter, Switzerland), whereas that to ceftazidime/avibactam and meropenem/vaborbactam by the E-test method (BioMérieux, Marcy l’Ètoile, France). Antimicrobial susceptibilities were interpreted according to the current EUCAST clinical breakpoints, with the ceftazidime/avibactam resistance clinical breakpoint being set at > 8 mg/L [29].

Patients underwent screening rectal swab according to the program of surveillance and control of healthcare-associated multidrug resistant Gram-negative infections of our institution that requires rectal screening swabs for new admissions and for inpatients on a weekly basis. The automated direct plating Wasp® instrument (Copan, Brescia, Italy) was used to inoculate the FecalSwab™ system (Copan, Brescia, Italy) on Brilliance CRE medium (Oxoid Ltd, Hampshire, UK).

### Statistical analysis

Summary descriptive statistics were presented as absolute counts (*n*) and percentage for categorical data and as median and interquartile range (IQR) for continuous variables. The Fisher’s exact test was used to compare proportions in the case of categorical variables between patients dying in-hospital and/or at 30 days following admission and those surviving, independently (i.e., 2 × 2 matrixes). In turn, for continuous features, the Mann–Whitney *U* test was employed. Exact *p*-values were reported in both cases. With the goal of dissecting the relative contribution of each variable for mortality while adjusting for every other feature (*Ceteris paribus*) and reducing the output variance, a random forest (RF) classifier was employed (1000 trees, 2/3^rd^ of the observations sampled at each iteration, seven variables randomly sampled at each split with replacement). Variable importance was ranked based on the GINI coefficient (average total decrease in node impurity), for in-hospital and 30-day all-cause mortality separately. For each individual patient, the model’s prediction was decomposed based on the contribution of the individual explanatory variables for that unique instance (all else held constant) and summarized in breakdown plots. For further scrutiny, this multivariate analysis was “optimized” using a forward stepping feature selection, settling on the simplest model among those with lower out-of-bag error rates. We allowed the process to converge on the same set of features for in-hospital and 30-day all-cause mortality, which consisted of kidney disease history (chronic and acute kidney injury following the ceftazidime/avibactam-resistant KPC-Kp BSI), recent renal replacement therapy history, the absolute Charlson Comorbidity index score, and whether cardiac surgery had been the reason for admission (Supplementary Figure S1). Features pertaining to hospitalization time/time from KPC-Kp BSI onset to discharge or death were deliberately left out of the model as these were not baseline predictors (i.e., could only be known after the fact) and were thus of questionable clinical relevance for future cases. Data analysis was performed in R version 4.2.2.

## Results

Over the study period, 362 KPC-Kp BSI episodes have been recorded. Among these, 38 (10.5%) were caused by ceftazidime/avibactam-resistant KPC-Kp strains and 37 events (from 37 individual patients) were included in this study (Supplementary Figure S2). Clinical features of patients included in the study are reported in Table 1. The median age was 59 years [IQR 52–69], 68% (*n* = 25) were men, 92% (*n* = 34) had been critically ill patients during the 30 days preceding the ceftazidime/avibactam-resistant KPC-Kp BSI onset, and 49% (*n* = 18) had been treated with ceftazidime/avibactam in the 90 days preceding the ceftazidime/avibactam-resistant KPC-Kp BSI. The median Charlson Comorbidity Index was 3 [IQR 1–5], and the comorbidities mainly observed were cardiovascular disease (35%, *n* = 13), diabetes (24%, *n* = 9), chronic respiratory disease (24%, *n* = 9), chronic kidney disease (Kidney Disease, Improving Global Outcomes 2012, stage 3A or worse, 24%, *n* = 9), and obesity (24%), with 24% (*n* = 9) of patients being solid-organ transplant recipients. Twenty-seven percent of patients underwent renal replacement therapy during the 30 days preceding the ceftazidime/avibactam-resistant KPC-Kp BSI onset. Eighty-nine percent of patients were KPC-Kp rectal carriers at the time of the ceftazidime/avibactam-resistant KPC-Kp BSI onset and in 12.1% (*n* = 4) of these a ceftazidime/avibactam-resistant KPC-Kp strain detected. The median total length-of-stay, length-of-stay before the ceftazidime/avibactam-resistant KPC-Kp BSI onset, and time from the ceftazidime/avibactam-resistant KPC-Kp BSI onset to discharge or death were 95 [IQR 52–150], 35 [IQR 20–72], and 44 [IQR 20–84] days, respectively. The main reasons for admission were sepsis (29.7%, *n* = 11), cardiac surgery (22%, *n* = 8), and other surgery (24%, *n* = 9). The main source of ceftazidime/avibactam-resistant KPC-Kp BSI was the respiratory tract (38%, *n* = 14), as 41% (*n* = 15) of cases were not secondary to infection from another site. The median Pitt Bacteremia and INCREMENT-CPE scores were 3 [IQR 1–5] and 6 [IQR 5–11], respectively. Ceftazidime/avibactam-resistant KPC-Kp strains included in the study were susceptible to meropenem, imipenem, gentamicin, and colistin in 62% (*n* = 23), 57% (*n* = 21), 51% (*n* = 19), and 46% (*n* = 17) of cases, respectively. Meropenem/vaborbactam showed higher activity (93%), but this activity was assessed on 14 strains only. Patients were mainly treated with combination of two antimicrobials (51%, *n* = 19), and 59% (*n* = 22) received active antibiotic therapy within 48 h of the ceftazidime/avibactam-resistant KPC-Kp BSI onset. Aminoglycosides- and meropenem/vaborbactam-including regimens were the treatment options mainly prescribed. Prolonged infusion of antibiotic ≥ 2 h and antibiotic dose adjusted for impaired renal function were carried out in 67.6% (*n* = 25) and 18.9% (*n* = 7) of cases, respectively. KPC-Kp infection relapse occurred in 18.9% (*n* = 7) of patients. In-hospital and 30-day all-cause mortality rates were 22% (*n* = 8) and 16.2% (*n* = 6), respectively.

**Table 1:** Clinical features of patients with ceftazidime/avibactam-resistant KPC-producing *Klebsiella pneumoniae* bloodstream infection

	*n* = 37
Patient characteristics	
Age, median [IQR] (years)	59 [52–69]
Male	68 (25)
Charlson Comorbidity Index, median [IQR]	3 [1–5]
Charlson Comorbidity Index ≥ 3	57 (21)
Diabetes	24 (9)
Cardiovascular disease	35 (13)
Chronic respiratory disease	24 (9)
Chronic kidney disease	24 (9)
Chronic liver disease	8.1 (3)
Solid-organ trasplant recipient	24 (9)
Neoplasia	13.5 (5)
Obesity	24 (9)
Renal replacement therapy 30 days preceding KPC-Kp BSI onset	27 (10)
KPC-Kp rectal carrier	89 (33)
Ceftazidime-avibactam resistant KPC-Kp rectal carrier	12.1 (4)
Previous exposure to ceftazidime/avibactam	49 (18)
ICU admission 30 days preceding KPC-Kp BSI onset	92 (34)
Total length of stay (days), median [IQR]	95 [52–150]
Hospital length of stay before KPC-Kp BSI (days), median [IQR]	35 [20–72]
Time from KPC-Kp BSI to discharge (days), median [IQR]	44 [20–84]
Reason for admission	
Sepsis	29.7 (11)
Cardiac surgery	22 (8)
Other surgery	24 (9)
Burn injury	8.1 (3)
Trauma	8.1 (3)
Stroke	5.4 (2)
Respiratory failure	2.7 (1)
Source of KPC-Kp BSI	
Primary KPC-Kp BSI	41 (15)
Respiratory tract	38 (14)
Intra-abdominal	11 (4)
Central venous catheter	5.4 (2)
Urinary tract	5.4 (2)
KPC-Kp BSI severity	
Pitt Bacteremia score, median [IQR]	3 [1–5]
INCREMENT-CPE score, median [IQR]	6 [5–11]
INCREMENT-CPE score ≥ 8	38 (14)
Shock	38 (14)
Invasive mechanical ventilation	51 (19)
Acute kidney injury	32 (12)
KPC-Kp susceptibility pattern	
Meropenem susceptible	62 (23)
Imipenem susceptible	57 (21)
Colistin susceptible	46 (17)
Fosfomycin susceptible	22 (8)
Amikacin susceptible	14 (5)
Gentamicin susceptible	51 (19)
Meropenem/vaborbactam susceptible	93 (13)
KPC-Kp BSI management	
Monotherapy	30 (11)
Combination of two antimicrobials	51 (19)
Combination of three or more antimicrobials	14 (5)
Active antibiotic therapy started within 48 h of KPC-Kp BSI onset	59 (22)
Carbapenem-including regimen	27 (10)
Colistin-including regimen	14 (5)
Tigecycline-including regimen	19 (7)
Aminoglycoside-including regimen	43 (16)
Fosfomycin-including regimen	22 (8)
Ceftazidime/avibactam-including regimen	16 (6)
Meropenem/vaborbactam-including regimen	35 (13)
Cefiderocol-including regimen	2.7 (1)
Prolonged infusion of antibiotic ≥ 2 h	67.6 (25)
Antibiotic dose adjusted for impaired renal function	18.9 (7)
Outcomes/complications	
Onset of superimposed and/or following KPC-Kp BSI treatment candidemia	8.1 (3)
KPC-Kp infection relapse	18.9 (7)
In-hospital mortality	22 (8)
30-day all-cause mortality	16.2 (6)

All data are shown as relative, %, and absolute (*n*) frequencies if not otherwise stated. Chronic kidney disease (Kidney Disease, Improving Global Outcomes 2012, stage 3A or worse)

*IQR* interquartile range, *ICU* intensive care unit, *KPC-Kp* KPC-producing *Klebsiella pneumoniae*, *BSI* bloodstream infection

**Fig. 1:**
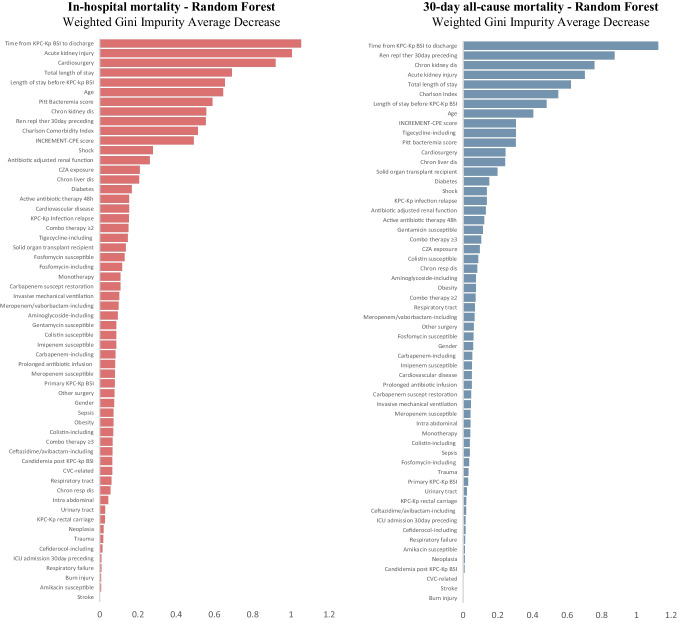
Variable importance (weighted average mean decrease Gini / node impurity) for the random forest classifiers in explanatory mode including every single variable under analysis for in-hospital (left) and 30-day all-cause mortality (right) among patients with ceftazidime/avibactam resistant KPC-producing *Klebsiella pneumoniae* bloodstream infection

**Fig. 2:**
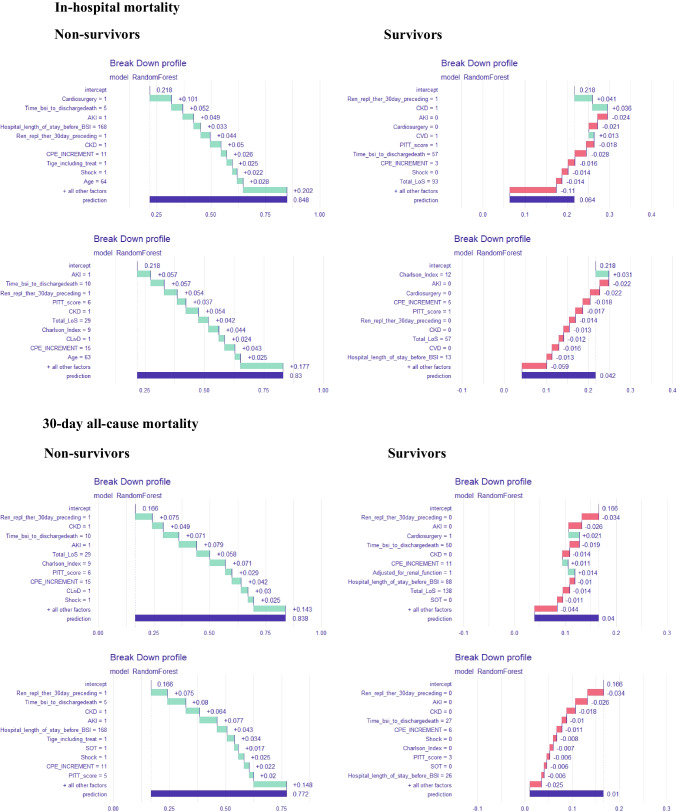
Breakdown plots with the contribution of each individual explanatory variable at the individual patient level. Two representative examples of both positive and negative cases for in-hospital and 30-day all-cause mortality in ceftazidime/avibactam-resistant KPC-producing *Klebsiella pneumoniae* bloodstream infections. Absolute values for continuous features and 0/1 (no/yes) for categorial ones. The intercept term represents the average predicted probability of death across the entire cohort, and subsequent entries display how that prediction changes based on the observed value of each explanatory variable (fixing the effect of every other variable). An individual feature contribution is influenced not only by its overall importance across the entire cohort but also by how much of an effect that variable had in explaining that specific patient’s outcome. Explanatory analysis, not intended for prediction/classification purposes

Patients who did not survive the hospital admission (Table 2) had significantly higher Charlson Comorbidity Indexes (*p*-value = 0.04), were more likely to suffer from chronic kidney disease (*p*-value = 0.01), had more often gone through renal replacement therapy during the 30 days preceding the ceftazidime/avibactam-resistant KPC-Kp BSI onset (*p*-value < 0.01), experienced a shorter time from the ceftazidime/avibactam-resistant KPC-Kp BSI onset to discharge or death (*p*-value = 0.02), were more likely to be admitted due to cardiac surgery (*p*-value < 0.01), and suffered from a more severe ceftazidime/avibactam-resistant KPC-Kp BSI (higher Pitt Bacteremia score, *p*-value 0.02; higher INCREMENT-CPE score, *p*-value = 0.03; more often developed shock and acute kidney injury, *p*-value = 0.03 and *p*-value < 0.01, respectively). Similarly, patients who did not survive past 30 days after the BSI (Table 2) had a significantly higher Charlson Comorbidity Index (*p*-value = 0.02), more often suffered from chronic kidney disease (*p*-value < 0.01), have more often gone through renal replacement therapy during the 30 days preceding the ceftazidime/avibactam-resistant KPC-Kp BSI onset (*p*-value < 0.01), experienced a shorter time from the ceftazidime/avibactam-resistant KPC-Kp BSI onset to discharge or death (*p*-value = 0.01), and suffered from a more severe ceftazidime/avibactam-resistant KPC-Kp BSI (more patients with INCREMENT-CPE score ≥ 8, *p*-value = 0.02; shock, *p*-value = 0.02; and acute kidney injury, *p*-value < 0.01). No statistically significant differences between survivors and non-survivors were noted for the KPC-Kp susceptibility pattern, KPC-Kp BSI antibiotic management, and complications following ceftazidime/avibactam-resistant KPC-Kp BSI. The overall distribution for the reported clinical scores is in Supplementary Figure S3. From all the features under consideration, the ensemble model employed to parse the true predictors from the data has consistently shown across both its simplest (data not shown) and most comprehensive **(**Fig. 1) versions that the presence of chronic kidney disease, acute kidney injury following the ceftazidime/avibactam-resistant KPC-Kp BSI, having recently gone through renal replacement therapy, having a higher Charlson Comorbidity Index, and being admitted for cardiac surgery were the driving features when it comes to accurately predicting whether a patient was likely to die due to ceftazidime/avibactam-resistant KPC-Kp BSI. Accordingly, these features were all positively associated with a higher chance of dying (both in-hospital and 30 days thereafter), while lower comorbidity scores, longer admission periods, and the absence of kidney injury/disease/cardiac surgery/renal replacement therapy have consistently reduced the likelihood of dying (Fig. 2).

**Table 2:** Features of survivors vs. non-survivors in ceftazidime/avibactam-resistant KPC-producing *Klebsiella pneumoniae* bloodstream infections

	In-hospital mortality			30-day mortality		
	Yes (x_med_ | *p̂*)	No (x_med_ | *p̂*)	*p*-value (Fisher’s | Mann–Whitney *U*)	Yes (x_med_ | *p̂*)	No (x_med_ | *p̂*)	*p*-value (Fisher’s | Mann–Whitney *U*)
Patient characteristics						
Age	63.5	56	0.28	63.5	58	0.42
Male	75%	66%	1	83%	65%	0.64
Charlson Comorbidity Index	5	2	0.04	5.5	3	0.02
Charlson Comorbidity Index ≥ 3	88%	48%	0.10	83%	52%	0.20
Diabetes	38%	21%	0.37	50%	19%	0.14
Cardiovascular disease	63%	28%	0.09	50%	32%	0.64
Chronic respiratory disease	25%	24%	1	33%	26%	0.61
Chronic kidney disease	63%	14%	0.01	83%	13%	< 0.01
Chronic liver disease	25%	3%	0.11	33%	3%	0.06
Solid organ transplant recipient	60%	21%	0.37	50%	19%	0.14
Neoplasia	-	14%	0.55	-	13%	1
Obesity	25%	24%	1	33%	26%	0.56
Renal replacement therapy 30 days preceding KPC-Kp BSI onset	75%	17%	< 0.01	100%	16%	< 0.01
KPC-Kp rectal carrier	100%	86%	0.55	100%	87%	1
Ceftazidime-avibactam resistant KPC-Kp rectal carrier	-	14%	0.55	-	13%	1
Previous exposure to ceftazidime-avibactam	63%	45%	0.44	67%	45%	0.40
ICU admission 30 days preceding KPC-Kp BSI onset	100%	90%	1	100%	90%	1
Total length of stay (days)	85.5	95	0.32	78	95	0.37
Hospital length of stay before KPC-Kp BSI (days)	31.5	36	0.76	31.5	36	0.68
Time from KPC-Kp BSI to discharge (days)	12	52	0.02	9	50	0.01
Reason for admission						
Sepsis	13%	34%	0.39	17%	32%	0.64
Cardiac surgery	63%	10%	< 0.01	50%	16%	0.10
Other surgery	25%	24%	1	33%	26%	0.61
Burn injury	-	10%	1	-	10%	1
Trauma	-	10%	1	-	10%	1
Stroke	-	7%	1	-	7%	1
Respiratory failure	-	3%	1	-	3%	1
Source of KPC-Kp BSI						
Primary KPC-Kp BSI	50%	38%	0.37	50%	39%	0.66
Respiratory tract	38%	38%	1	50%	55%	0.65
Intra-abdominal	-	14%	0.55	-	13%	1
Central venous catheter	13%	3%	0.39	-	7%	1
Urinary tract	-	7%	1	-	7%	1
KPC-Kp BSI severity						
Pitt Bacteremia score	5	2	0.02	5	2	0.06
INCREMENT-CPE score	11	6	0.03	11	6	0.05
INCREMENT-CPE score ≥ 8	75%	28%	0.03	83%	29%	0.02
Shock	75%	28%	0.03	83%	29%	0.02
Invasive mechanical ventilation	75%	45%	0.23	67%	48%	0.65
Acute kidney injury	88%	17%	< 0.01	100%	19%	< 0.01
KPC-Kp susceptibility pattern						
Meropenem susceptible	50%	66%	0.44	50%	64%	0.65
Imipenem susceptible	50%	59%	0.70	50%	58%	1
Colistin susceptible	50%	45%	1	67%	42%	0.38
Fosfomycin susceptible	-	28%	0.15	-	26%	0.30
Amikacin susceptible	-	17%	0.56	-	16%	0.56
Gentamicin susceptible	63%	48%	0.69	67%	48%	0.65
Meropenem/vaborbactam susceptible	25%	38%	0.68	33%	35%	1
KPC-Kp BSI management						
Active antibiotic therapy started within 48 h of KPC-Kp BSI onset	75%	55%	0.43	67%	58%	1
Monotherapy	13%	63%	0.39	17%	32%	0.64
Combination of two antimicrobials	63%	48%	0.69	50%	52%	1
Combination of three or more antimicrobials	13%	14%	1	17%	13%	1
Carbapenem-including regimen	25%	28%	1	17%	29%	1
Colistin-including regimen	13%	14%	1	17%	13%	1
Tigecycline-including regimen	38%	14%	0.15	50%	13%	0.06
Aminoglycoside-including regimen	38%	45%	1	50%	42%	1
Fosfomycin-including regimen	13%	24%	0.65	-	26%	0.30
Ceftazidime/avibactam-including regimen	25%	14%	0.59	17%	16%	1
Meropenem/vaborbactam-including regimen	38%	53%	1	33%	35%	1
Cefiderocol-including regimen	-	3%	1	-	3%	1
Prolonged infusion of antibiotic ≥ 2 h	75%	66%	1	67%	68%	1
Antibiotic dose adjusted for impaired renal function	38%	14%	1	33%	16%	0.31
Outcomes/complications						
Onset of superimposed and/or following KPC-Kp BSI treatment candidemia	13%	7%	0.52	-	10%	1
KPC-Kp infection relapse	13%	21%	1	-	23%	0.57

Data shown as sample median (**med(x)) or sample proportion (p̂) for each group.** Comparisons performed using the Fisher’s exact test for categorial features and the Mann–Whitney* U* Test for continuous variables. Bold values denote statistical significance at the 5% significance level (*p* < 0.05). Chronic kidney disease (Kidney Disease, Improving Global Outcomes 2012, stage 3A or worse)

*ICU* intensive care unit, *KPC-Kp* KPC-producing *Klebsiella pneumoniae*, *BSI* bloodstream infection

## Discussion

This work represents the largest study published to date on factors associated with mortality in patients hospitalized due to ceftazidime/avibactam-resistant KPC-Kp BSI, shedding some light over the associated mortality patterns 5 years after the introduction of this antibiotic into clinical practice which has nonetheless improved clinical outcomes in KPC-Kp infections [2–8]. Ceftazidime/avibactam resistance in KPC-Kp BSI effortlessly emerged in our highly KPC-Kp endemic area. The ceftazidime/avibactam-resistant isolates revealed susceptibility restoration to at least one carbapenem in more than 60% of cases. The overall mortality across the entire cohort was remarkable for both in-hospital and 30-day thereafter. Perhaps not surprising, patients who ended up dying suffered from more baseline comorbidities and experienced a more severe ceftazidime/avibactam-resistant KPC-Kp BSI presentation (i.e., both the Pitt Bacteremia and INCREMENT-CPE scores were significantly higher). Among such comorbidities, kidney disease (chronic—KDIGO stage 3A or worse—and acute kidney injury following the ceftazidime/avibactam-resistant KPC-Kp BSI onset) stood out and one of the strongest predictive factors. In parallel, renal replacement therapy was also much more often observed in patients failing to survive the ceftazidime/avibactam-resistant KPC-Kp BSI. Notably, of all the reasons for admission, cardiac surgery was the one most strongly associated with KPC-Kp BSI mortality. In contrast, the KPC-Kp susceptibility pattern, history of previous ceftazidime/avibactam treatment, the onset of concomitant and/or superimposed candidemia, and developing a KPC-Kp infection relapse presented a non-negligible yet much more modest role in predicting patient mortality.

In the light of the excellent clinical results obtained [3–9], the judicious prescription of ceftazidime/avibactam and the implementation of a surveillance system dedicated to resistance emergence detection should be aimed at. For the time being, KPC-Kp resistance to ceftazidime/avibactam is not perceived as a problem at scale, being deemed a rather uncommon phenomenon [11]. Accordingly, during the first wave of the SARS-CoV-2 pandemic, a Southern Italian multicenter surveillance study reported a single case of ceftazidime/avibactam-resistant KPC-Kp throughout an entire 6-month carbapenem-non-susceptible Enterobacterales isolates collection period [30]. In contrast, although an outbreak occurred in a COVID-19 ICU [12], the prevalence of ceftazidime/avibactam resistance in KPC-Kp BSI isolates in our center has remained stable at around 10% [31] reaching values more similar to those published by Gaibani et al*.* for the period 2018–2020 in another Northern Italian center [32].

The susceptibility patterns of ceftazidime/avibactam-resistant KPC-Kp strains herein reported are in line with other reports [11], whereby a susceptibility restoration phenomenon to at least one carbapenem (despite no association with previous ceftazidime/avibactam exposure) was observed. The fact that ceftazidime/avibactam-resistant KPC-Kp isolates with reduced carbapenemase activity are the most widespread is not inconsequential. From a diagnostic standpoint, these may often go undetected, especially by the phenotypic methods most commonly used in hospital surveillance systems [19–22]. From a therapeutic perspective, the effectiveness of pre-emptive therapy with ceftazidime/avibactam might be limited when ceftazidime/avibactam-resistant KPC variants start to circulate in a hospital, even considering that ceftazidime/avibactam exposure induces resistance development more frequently in the blood and respiratory tract than in the rectum [33], highlighting the need to update the diagnostic protocols of surveillance cultures [12].

Mortality rates of patients with ceftazidime/avibactam-resistant KPC-Kp infections have been estimated in a review paper by Di Bella et al*.* at around 37% [11], moving us backwards all the way to when ceftazidime/avibactam was not yet available as therapeutic option. In the present study, both in-hospital and 30-day all-cause mortality rates were lower than the aforementioned, being closer to those reported among patients suffering from ceftazidime/avibactam-susceptible KPC-Kp infections and treated with it [2, 6].

Notably, even though most of the ceftazidime/avibactam-resistant KPC-Kp strains isolated for the purposes of this study had restored susceptibility to carbapenems and most patients had been treated within 48 h of the BSI onset, no single therapeutic regimen could be pinpointed as factor associated with reduced mortality in this situation. In all likelihood, data is too sparse in the present study in order to confidently dissect these so that whether such mortality-reducing therapeutic regimens exist remains an open question.

The present study is limited in its retrospective nature, because it has been conducted at a single center, and due to the limited number of cases for each combination of features of interest. Nonetheless, this study emphasizes the importance of epidemiological surveillance with the goal of keeping resistance to ceftazidime/avibactam at bay and highlights the clinical features that might be relevant for mortality. Whether these and other features are actionable remains to be seen, especially since all the important predictors herein highlighted are not modifiable.

Current efforts should be focused on the early identification of ceftazidime/avibactam-resistant KPC-Kp and respective susceptibility patterns to avoid rounds of failed treatment attempts, on good coordination and implementation of a quick and efficient surveillance system. The identification of how best to proceed at the individual patient level in the face of a ceftazidime/avibactam-resistant KPC-Kp BSI as identified may prove useful for individual patient risk stratification.

### Supplementary Information

Below is the link to the electronic supplementary material.

Supplementary file1 (DOCX 97 KB)

Supplementary file2 (DOCX 26 KB)

Supplementary file3 (DOCX 155 KB)

## Data Availability

The authors confirm that the data supporting the findings of this study are available within the article.
