# Supplementary material for: Prevalence and mortality of ceftazidime/avibactam-resistant KPC-producing Klebsiella pneumoniae bloodstream infections (2018–2022)
Source: Eur J Clin Microbiol Infect Dis. 2023 Nov 21;43(1):155–66. doi: 10.1007/s10096-023-04712-8 (PMC10774640; doi:10.1007/s10096-023-04712-8)
Supplement: Supplementary file 1 — Supplementary file1 (DOCX 97 KB) [file 10096_2023_4712_MOESM1_ESM.docx]

**30-day all-cause mortality**

**In-hospital mortality**

**
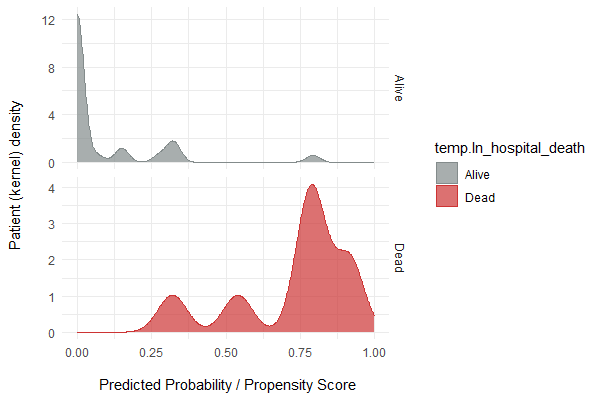

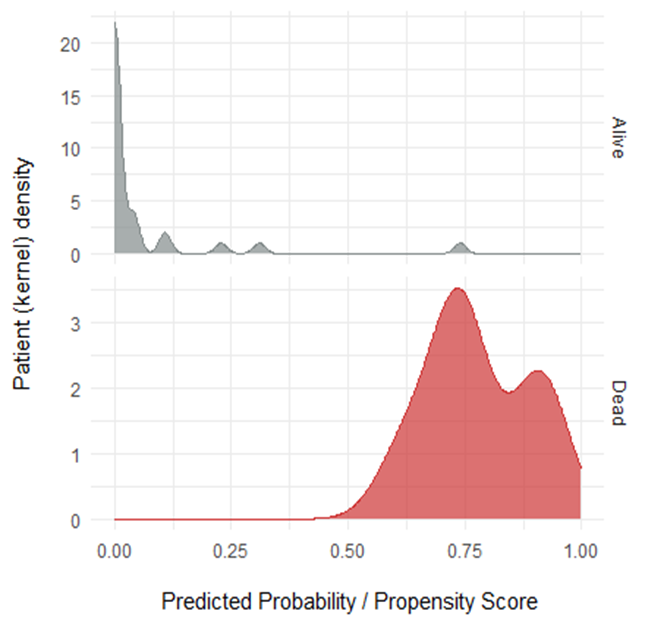
**

**Supplementary Figure S1 -** Estimated probability of dying / propensity scores for patients with ceftazidime/avibactam-resistant KPC-producing *Klebsiella pneumoniae* bloodstream infection when employing a random forest classifier consisting of chronic kidney disease, acute kidney injury following the ceftazidime/avibactam-resistant KPC-Kp bloodstream infection, recent renal replacement therapy history, the absolute Charlson Comorbidity Index score and whether cardiac surgery had been the reason for admission. This reduced-feature RF classifier provided an accuracy of 89% (75% F1 score, given the unbalanced nature of the dataset) in predicting in-hospital mortality, and an accuracy of 97% (93% F1 score, idem) in predicting 30-day all-cause mortality using a hold-out validation set.
