# Supplementary material for: Prevalence and mortality of ceftazidime/avibactam-resistant KPC-producing Klebsiella pneumoniae bloodstream infections (2018–2022)
Source: Eur J Clin Microbiol Infect Dis. 2023 Nov 21;43(1):155–66. doi: 10.1007/s10096-023-04712-8 (PMC10774640; doi:10.1007/s10096-023-04712-8)
Supplement: Supplementary file 2 — Supplementary file2 (DOCX 26 KB) [file 10096_2023_4712_MOESM2_ESM.docx]

**Supplementary Figure S2**. Flowchart of the study (2018-2022).

KPC-Kp bloodstream infection episodes

n=362

Ceftazidime/avibactam susceptible (MIC ≤ 8 mg/L) KPC-Kp bloodstream infection episodes excluded (n=322)

No medical records excluded (n=1)

Multi-carbapenemases producers (KPC+VIM) bloodstream infection episodes excluded (n=2)

Ceftazidime/avibactam resistant KPC-Kp bloodstream infection episodes included

n=37

Ceftazidime/avibactam resistant KPC-Kp bloodstream infection episodes

n=38

Ceftazidime/avibactam resistant KPC-Kp bloodstream infection episodes

n=40
