# Supplementary material for: Prevalence and mortality of ceftazidime/avibactam-resistant KPC-producing Klebsiella pneumoniae bloodstream infections (2018–2022)
Source: Eur J Clin Microbiol Infect Dis. 2023 Nov 21;43(1):155–66. doi: 10.1007/s10096-023-04712-8 (PMC10774640; doi:10.1007/s10096-023-04712-8)
Supplement: Supplementary file 3 — Supplementary file3 (DOCX 155 KB) [file 10096_2023_4712_MOESM3_ESM.docx]

**In-hospital mortality**


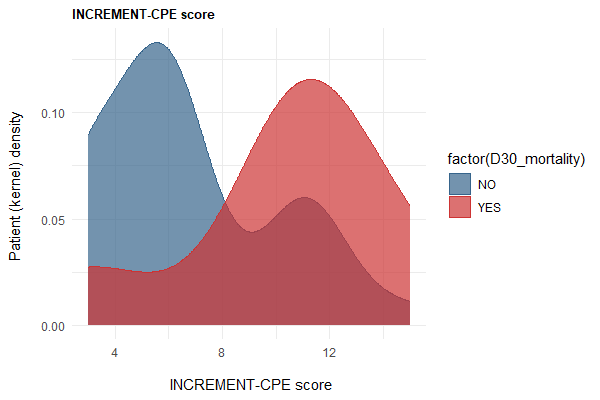

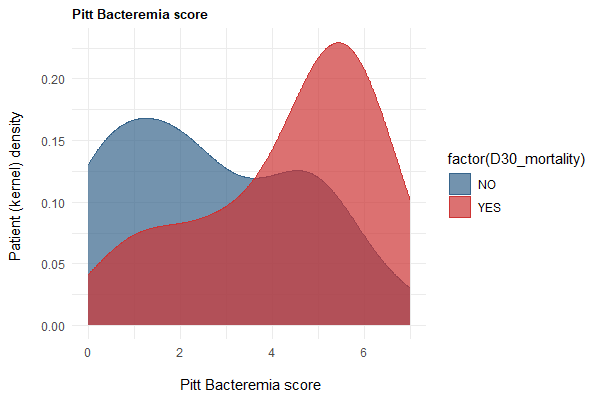

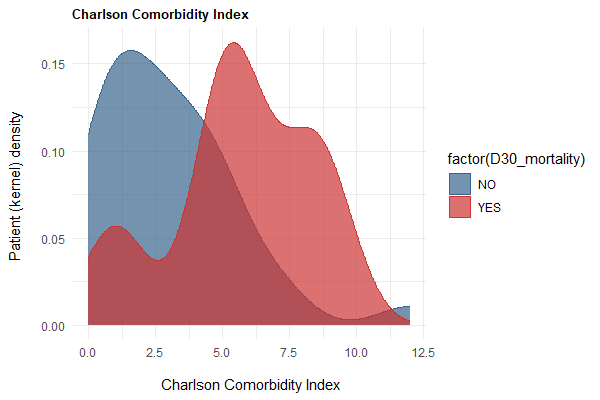


**30-day all-cause mortality**


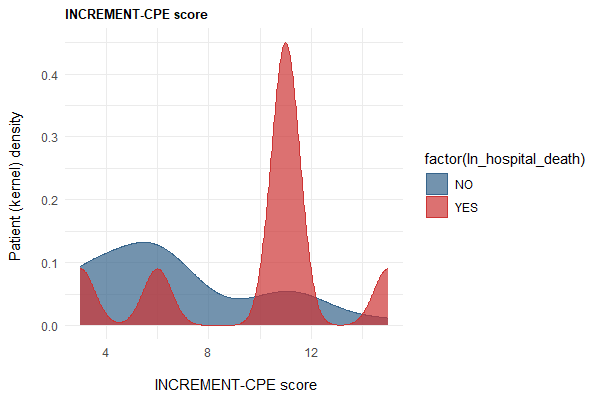

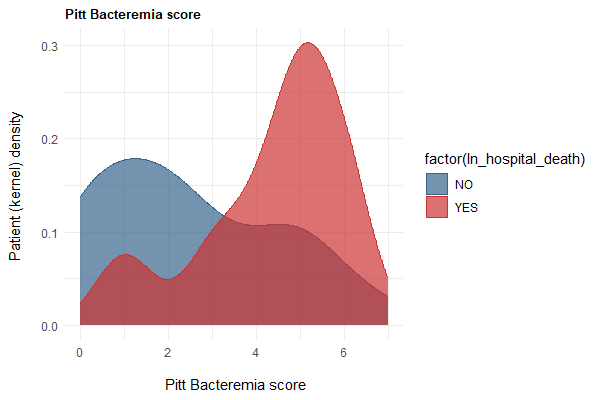

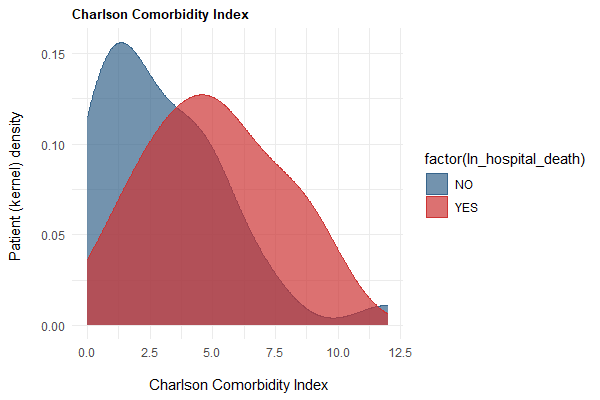


**Supplementary Figure S3**. Gaussian smoothing kernel density estimates distribution for the Charlson Comorbidity Index, Pitt Bacteremia score and INCREMENT-CPE score as a function of the in-hospital and 30-day all-cause mortality status among patients with ceftazidime/avibactam-resistant KPC-producing *Klebsiella pneumoniae* bloodstream infection (blue: survivors; red: non-survivors).
